# Genome-wide in silico characterization, validation, and cross-species transferability of microsatellite markers in Mallard and Muscovy ducks

**DOI:** 10.1186/s43141-023-00556-z

**Published:** 2023-10-19

**Authors:** Hosam Safaa, Rawan Khaled, Suzy Isaac, Rofida Mostafa, Mohamed Ragab, Dalia A. A. Elsayed, Mostafa Helal

**Affiliations:** 1 Department of Biology, College of Science, University of Bisha, P.O. Box 551, 61922 Bisha, Saudi Arabia; 2 Department of Animal Production, Faculty of Agriculture, Cairo University, Giza, 12613 Egypt; 3 Biotechnology Program, Faculty of Agriculture, Cairo University, Giza, 12613 Egypt; 4 Poultry Production Department, Faculty of Agriculture, Kafrelsheikh University, Kafrelsheikh, Egypt; 5 Animal Breeding and Genetics Department, National Institute for Agricultural and Food Research and Technology (INIA), 28040 Madrid, Spain; 6 Department of Poultry Breeding, Agriculture Research Center, Animal Production Research Institute, Dokki, Giza, Egypt

**Keywords:** In silico, Microsatellite, SSR, Muscovy duck, Mallard duck

## Abstract

**Background:**

Microsatellites are important markers for livestock including ducks. The development of microsatellites is expensive and labor-intensive. Meanwhile, the in silico approach for mining for microsatellites became a practicable alternative. Therefore, the current study aimed at comparing whole-genome and chromosome-wise microsatellite mining approaches in Muscovy and Mallard ducks and testing the transferability of markers between them. The GMATA software was used for the in silico study, and validation was performed using 26 primers.

**Results:**

The total number of the detected microsatellites using chromosome-wise was 250,053 and 226,417 loci compared to 260,059 and 238,462 loci using whole genome in Mallards and Muscovies. The frequencies of different motifs had similar patterns using the two approaches. Dinucleotide motifs were predominant (> 50%) in both Mallards and Muscovies. The amplification of the genomes revealed an average number of alleles of 5.08 and 4.96 in Mallards and Muscovies. One locus was monographic in Mallards, and two were monomorphic in Muscovies. The average expected heterozygosity was higher in Muscovy than in Mallards (0.45 vs. 0.43) with no significant difference between the two primer sets, which indicated the usefulness of cross-species amplification of different primers.

**Conclusion:**

The current study developed a whole-genome SSR panel for ducks for the first time, and the results could prove that using chromosome-wise mining did not generate different results compared to the whole-genome approach.

## Background

Ducks are unique poultry species, as they fall into two species which are Mallard (*Anas platyrhynchos*) and Muscovy (*Cairina moschata*) ducks. Mallard ducks are more common than Muscovy and are considered to be social animals that like to live in groups that differ in size [1, 2]. They are also considered the largest and most widespread of waterfowl and the second-largest poultry species [3]. The first comparative and cytogenetic map of the ducks was published in the year 1966 for Mallard ducks [4], where the researchers postulated that the diploid chromosome number of ducks was 80 unlike 78 chromosomes for chickens [5]. Recently, deep analyses for duck chromosomes were performed [6], when the researchers successfully delineated 19 linkage groups wherein 115 microsatellite markers were placed for defining the duck genome. Thus, a sex-corrected map spanning 1353.3-cm length, with a mean interval distance of 15.04 cm per marker, was established. These efforts were followed by forming a consortium for detailed sequencing of the duck genome, where they sequenced the genome of a female Beijing duck by generating 77 Gb of paired-end reads amounting to 64-fold coverage of the whole genome [6].

From the repetitive sequences are the microsatellites, which are simple sequence repeats (SSR). The term “microsatellites” was developed in 1989 during research on the (TG)n gene of cardiac actin [7]. They are DNA motifs that are repeated about 5–50 times which are found thousands of times on the chromosome. The creation of microsatellites is non-random, with distinct differences among mechanisms that stimulated the genes for SSRs, which included insertions, deletions, recombination, repair, transpositions, horizontal gene transfer, and replication slippage. Microsatellites have been detected within the genomes of all eukaryotes [8]. Microsatellites are the most adaptable molecular markers, which are utilized to identify a specific molecular sequence in a pool of unknown DNA. This helps in determining their connections and evolutionary links in closely related genomes. Repeated repetitions are becoming one of the most popular genetic probes. They are currently popular in molecular genetics, biotechnology, and evolutionary biology [9]. They are polymorphic which means they have many potential alleles at different places on the chromosome; also, they can be inherited easily. Various techniques have been established to evaluate DNA polymorphism by measuring genetic diversity in situ. Consequently, it is easy to trace the fingerprints of all the organisms by examining molecular markers of DNA involved in determining the inherited characters and evolutionary history in a phyletic lineage [10, 11]. The direct applications for developing new microsatellites are not limited. For instance, the chromosome-level assembly of the Muscovy duck genome offers valuable information on the susceptibility of fatty liver [12]. Also, the comparison of markers in different duck species can provide insights into the molecular basis of disease susceptibility [13]. The evolutionary studies in ducks also benefited from the applications of microsatellite markers [14, 15]. The characterization of microsatellite repeats and their variation in ducks could facilitate their use as genetic markers and consequently allow breeding strategies that focus on the transfer of markers from one breed to another to be applied. Also, it helps in the identification of genes/QTLs controlling economic traits, making them more useful in studies involving marker-trait association, QTL mapping, and genetic diversity analysis.

Therefore, the current study aimed at in silico analysis of the whole-genome sequence of both Mallard and Muscovy ducks, for mining the genome to generate a panel of microsatellite loci. The study also compares whole-genome and chromosome-wise mining approaches.

## Methods

### Ethical approval

The genotyping and validation procedures were approved by the Institutional Animal Care and Use Committee at Cairo University (CU-IACUC), with the approval number of CU/I/F/32/23.

### Sequence data source

The reference genome sequences were downloaded from the National Center for Biotechnology Information (NCBI); the sequence of common ducks was for the Z2 breed of Pekin duck (accession no.: GCA_015476345.1). All available chromosome sequences were subjected to analysis. For Muscovy ducks, the reference genome sequence of the CM-2020 isolate (accession no.: GCA_018104995.1) was used.

### In silico mining of whole-genome-wide SSRs

The genome sequences were analyzed in two approaches. The first one was the whole-genome approach, where a single FASTA format file for whole-genome sequence for each of Mallard and Muscovy ducks was downloaded and subjected to the analysis. The second approach was chromosome-by-chromosome analysis, in which chromosome-wise FASTA format files were downloaded and analyzed. The analyzed genome sizes were 1189 and 1119 Mbp for Mallard and Muscovy ducks, respectively. For Mallard ducks, the chromosomal sequences were available for all the chromosomes from 1 to 33 except chromosomes 30 and 32, which were not available on the NCBI webpage. The analysis also included sex chromosomes (W and Z) and mitochondrial DNA sequences. For Muscovy ducks, the available sequences were for the chromosomes from 1 to 29, as well as the Z chromosome.

These sequences were mined for SSRs using Genome-wide Microsatellite Analyzing Tool (GMATA) software package version 2.1 [16]. The choice of GMATA was due to its ability to analyze large sequences by chunking them into lesser fragments to increase mining speeds. The criteria used for repeat identification were set to minimum and maximum repeat lengths of 2 and 6, respectively, and minimum repeat time of five for di-, tri-, tetra-, penta-, and hexanucleotide motifs. Primer pairs were thereafter developed using primer3 included in the GMATA package, where the SSR file (GMATA output) along with the sequence file was used as input files for designing the primers. The parameters were set to amplicons that ranged between 120 and 400 bp, with a flanking sequence length of 400 bp, and an average annealing temperature of 60 °C.

For comparisons, relative abundance and relative density were calculated. Relative abundance was calculated as the number of SSRs per Mb of the sequence analyzed, and relative density was calculated as the length (in bp) of SSRs per Mb of the sequence analyzed.

### Validation of microsatellites and PCR analysis

A total of 26 primer pairs (13 per duck species, Table 1) were randomly chosen across the genome and designed to validate the detected loci and the developed primers. Validation was performed using polymerase chain reaction (PCR). Individual blood samples were collected from twenty birds of both sexes of each of Muscovy and Mallard ducks. DNA was extracted using WizPrep genomic DNA kit (Wizbiosolutions Inc., South Korea) according to the manufacturer’s instructions. The PCR reaction volume was adjusted to 20 μl, including 2 μl of template DNA (~ 30–50 ng), 1 μl of each of the forward and reverse primers, 10 μl of master mix, and 6 μl of nuclease-free water. The amplification program started with the initial denaturation step (95 °C for 5 min), followed by 35 cycles of denaturation (30 s at 95 °C), annealing (54 s at 56–63 °C), and elongation (30 s at 72 °C), and the program was closed by a final extension step at 72 °C for 10 min. The PCR products were then separated by electrophoresis using 8% non-denaturing polyacrylamide gel. The genomic parameters, including the number of alleles, minor allele frequency, expected heterozygosity, and number of effective alleles, were estimated using GenAlex [17] and POPGENE [18] software packages. Polymorphic information content (PIC) was calculated for each marker using Gene-Calc online platform (https://gene-calc.pl/pic). The generated results were compared using the Student’s *t*-test.

**Table 1:** Primer sequence, polymorphism, observed allele range, and number of alleles/locus of the selected loci

Marker ID	Species	Primer		TM	Fragment size
		Forward	Reverse		
MK22	Muscovy	GACGAGGACAGCAGCTTGA	TAACTGGAAGCCATGCACAG	60.1	353
MK37651	Muscovy	CCCCAACACACACTACCACA	AATACCAGGATGCCAACTGC	60.1	246
MK37669	Muscovy	ATGCATACACTGCTGCTTGG	CCAGGAAACAAAATGGGAAA	59.8	367
MK37680	Muscovy	TCCATTCAATCACCAGCTCA	GCCATCAAAAGATCCTCCAG	59.9	400
MK19696	Muscovy	TTGGTGGTGAATCATGTGCT	GTCCTCTGTGATGGGTGCTT	60.0	310
MK19508	Muscovy	GCCAGAATTGTTTCTGCATGT	GCTGATGCACAGTCATTGGT	59.9	355
MK19002	Muscovy	GAGGAGGAGGAGGGAGAGAG	GGCTTTGTGTGTGTGTGTGG	60.3	225
MK38645	Muscovy	CTGGCTGTGGAGACGGTAAT	AGGATTCATGCTGCTGCTTT	60.1	183
MK33624	Muscovy	TCCAGAAGAAAAGGGGATGA	TTCCCAAAGGAATTTTGCTG	59.8	225
MK31272	Muscovy	CCTGGCTAAGCAGCTGAAAA	TGCTTTGTGAAGTTGATGCAG	60.3	210
MK26001	Muscovy	ATGGCAGCAGGAGATAAGGA	CACCCCGAAGTAAACACCAT	59.8	181
MK37601	Muscovy	CTGCATAAGCCAATGCTGAA	TTCTTTCCCTTTCCCTTTCC	59.7	171
MK46014	Mallard	GACGAGGACAGCAGCTTGA	TAACTGGAAGCCATGCACAG	60.1	356
MK4647	Mallard	CCCCAACACACACTACCACA	AATACCAGGATGCCAACTGC	60.1	269
MK5039	Mallard	CCAGGAAACAAAATGGGAAA	ATGCATACACTGCTGCTTGG	59.8	347
MK4624	Mallard	GCCATCAAAAGATCCTCCAG	TCCATTCAATCACCAGCTCA	59.9	400
MK24903	Mallard	GTCCTCTGTGATGGGTGCTT	TTGGTGGTGAATCATGTGCT	60.0	304
MK25130	Mallard	GCTGATGCACAGTCATTGGT	GCCAGAATTGTTTCTGCATGT	59.9	343
MK25365	Mallard	GGCTTTGTGTGTGTGTGTGG	GAGGAGGAGGAGGGAGAGAG	60.3	231
MK3631	Mallard	AGGATTCATGCTGCTGCTTT	CTGGCTGTGGAGACGGTAAT	60.1	191
MK8745	Mallard	TTCCCAAAGGAATTTTGCTG	TCCAGAAGAAAAGGGGATGA	59.8	235
MK11161	Mallard	ATGCTTCGTGAAGTTGATGC	CCTGGCTAAGCAGCTGAAAA	59.8	210
MK17028	Mallard	CACCCCGAAGTAAACACCAT	ATGGCAGCAGGAGATAAGGA	59.8	178
MK15735	Mallard	GTTTCAGCCCGAAGAAACTG	TTCTTTCCCTTTCCCTTTCC	59.7	398

## Results

**Fig. 1:**
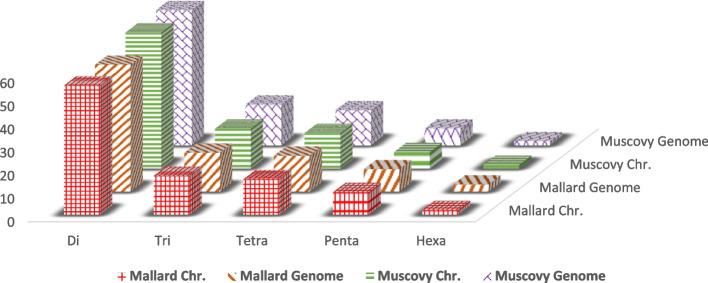
Frequency of different SSR motifs developed in the genomes of Mallard and Muscovy ducks using genome-wise and whole-genome mining approaches

In the current study, we used two approaches for mining SSR, the chromosome-wise approach generated fewer microsatellites than whole-genome analysis in the two duck species, where the total number of the detected microsatellites using chromosome wise was 250,053 and 226,417 loci compared to 260,059 and 238,462 loci using whole genome in Mallard and Muscovy ducks, respectively. Also, the frequencies of different motifs had similar patterns using the two approaches as shown in Fig. 1.

### Chromosome-wise SSR mining

The detected motif types and frequencies for each chromosome are presented in Table 2 for Mallards and Table 3 for Muscovy ducks. The dinucleotide was predominant in both Mallards and Muscovies, where it accounted for 55.89 and 58.75% of the total detected motifs in the two species, respectively. The trinucleotide ranked second at 16.90 and 17.14% for Mallards and Muscovies, respectively. However, hexanucleotide motifs were the least and accounted for 2.07 and 1.66 for Mallard and Muscovy ducks, respectively.

**Table 2:** Frequency and distribution of the microsatellite loci detected for each chromosome of Mallard duck genome

Chr	Motif (*k*-mer)				
	Dinucleotide	Trinucleotide	Tetranucleotide	Pentanucleotide	Hexanucleotide
1	30,089 (52.8)	9155 (16.1)	10,139 (17.8)	6298 (11.1)	1271 (2.2)
2	23,208 (53.5)	7062 (16.3)	7365 (16.99)	4842 (11.2)	885 (2.04)
3	16,094 (54.5)	4726 (15.99)	4901 (16.6)	3325 (11.3)	507 (1.7)
4	10,074 (61.15)	2599 (15.78)	2306 (13.99)	1261 (7.66)	234 (1.42)
5	7841 (62.22)	2031 (16.12)	1614 (12.8)	971 (7.7)	145 (1.15)
6	4284 (66.96)	1020 (15.9)	727 (11.36)	309 (4.8)	58 (0.9)
7	4678 (66.2)	1118 (15.81)	811 (11.47)	395 (5.59)	68 (0.96)
8	3726 (65.15)	908 (15.88)	665 (11.63)	318 (5.56)	102 (1.78)
9	2692 (63.6)	754 (17.81)	511 (12.1)	228 (5.39)	48 (1.13)
10	2337 (62.42)	674 (18)	476 (12.71)	215 (5.74)	42 (1.12)
11	2124 (63.23)	625 (18.6)	382 (11.37)	195 (5.8)	33 (0.98)
12	2282 (63.74)	628 (17.54)	383 (10.7)	222 (6.2)	65 (1.82)
13	2395 (67.75)	635 (17.96)	310 (8.8)	163 (4.61)	32 (0.9)
14	1900 (56.22)	651 (19.27)	340 (10.06)	233 (6.9)	255 (7.55)
15	1672 (66.5)	434 (17.3)	234 (9.3)	140 (5.6)	34 (1.35)
16	1500 (56.86)	447 (16.95)	329 (12.47)	124 (4.7)	238 (9.02)
17	110 (40.3)	64 (23.4)	44 (16.12)	47 (17.22)	8 (2.93)
18	1007(62.98)	347 (21.70)	129 (8.07)	94 (5.88)	22 (1.38)
19	1061 (58.5)	412 (22.72)	171 (9.4)	134 (7.39)	35 (1.9)
20	935 (62.8)	325 (21.84)	137 (9.2)	63 (4.2)	28 (1.88)
21	1508 (61.58)	469 (19.15)	246 (10.05)	189 (7.72)	37 (1.5)
22	735 (64.99)	251 (22.19)	83 (7.34)	44 (3.89)	18 (1.59)
23	430 (59.6)	168 (23.3)	61 (8.46)	50 (6.9)	12 (1.66)
24	607 (53.06)	342 (29.9)	85 (7.43)	93 (8.13)	17 (1.49)
25	639 (64.68)	228 (23.08)	62 (6.28)	42 (4.25)	17 (1.72)
26	201 (36.61)	193 (35.16)	41 (7.47)	39 (7.1)	75 (13.66)
27	486 (54.3)	293 (32.74)	55 (6.15)	45 (5.03)	16 (1.79)
28	615 (51.72)	273 (22.96)	128 (10.77)	146 (12.28)	27 (2.27)
29	434 (54.66)	228 (28.72)	51 (6.42)	63 (7.94)	18 (2.27)
31	12 (30.77)	20 (51.28)	1 (2.56)	6 (15.39)	0
33	4 (13.79)	19 (65.52)	2 (6.9)	1 (3.45)	3 (10.34)
W	1097 (48.45)	750 (33.1)	213 (9.4)	146 (6.45)	58 (2.56)
Z	12,982 (47.1)	4402 (15.96)	5417 (19.7)	4008 (14.5)	766 (2.78)
MT	1 (100)	0	0	0	0
Total	139,760	42,251	38,419	24,449	5174
Percentage	55.89%	16.90%	15.36%	9.78%	2.07%

**Table 3:** Frequency and distribution of the microsatellite loci detected for each chromosome of Muscovy duck genome

Chr	Motif (*k*-mer)				
	Dinucleotide	Trinucleotide	Tetranucleotide	Pentanucleotide	Hexanucleotide
1	28,792 (55.56)	8571 (16.54)	9141 (17.64)	4349 (8.39)	973 (1.88)
2	21,824 (56.53)	6498 (16.83)	6513 (16.87)	3086 (7.99)	688 (1.78)
3	16,156 (57.83)	4687 (16.78)	4482 (16.04)	2123 (7.6)	488 (1.75)
4	10,203 (62.1)	2728 (16.6)	2396 (14.58)	910 (5.54)	193 (1.18)
5	8804 (64.59)	2205 (16.18)	1693 (12.42)	756 (5.55)	173 (1.27)
6	2351 (65.34)	601 (16.7)	424 (11.78)	186 (5.17)	36 (1.00)
7	3413 (67.92)	802 (15.96)	555 (11.05)	214 (4.26)	41 (0.82)
8	5675 (67.83)	1298 (15.52)	954 (11.40)	352 (4.21)	87 (1.04)
9	3068 (65.95)	742 (15.95)	559 (12.02)	245 (5.27)	38 (0.82)
10	2147 (67.2)	544 (17.03)	349 (10.92)	135 (4.23)	20 (0.63)
11	1513 (63.79)	521 (21.97)	192 (8.09)	120 (5.06)	26 (1.10)
12	1513 (66.1)	394 (17.21)	282 (12.32)	79 (3.45)	21 (0.92)
13	2377 (69.08)	595 (17.29)	330 (9.59)	122 (3.55)	17 (0.5)
14	1574 (61.9)	524 (20.61)	262 (10.30)	155 (6.1)	28 (1.1)
15	1668 (66.99)	446 (17.91)	237 (9.52)	111 (4.46)	28 (1.13)
16	1335 (67.8)	371 (18.84)	158 (8.02)	84 (4.27)	21 (1.07)
17	55 (46.61)	38 (32.2)	12 (10.17)	9 (7.63)	4 (3.39)
18	1010 (63.4)	365 (22.91)	133 (8.35)	67 (4.21)	18 (1.13)
19	947 (63.94)	316 (21.34)	127 (8.58)	73 (4.93)	18 (1.22)
20	629 (56.87)	308 (27.85)	91 (8.23)	62 (5.61)	16 (1.45)
21	1514 (63.83)	412 (17.37)	265 (11.17)	142 (5.99)	39 (1.64)
22	718 (66.36)	238 (22)	76 (7.02)	39 (3.6)	11 (1.02)
23	159 (56.79)	93 (33.21)	9 (3.21)	14 (5)	5 (1.79)
24	523 (59.57)	219 (24.94)	78 (8.88)	44 (5.01)	14 (1.59)
25	629 (65.45)	227 (23.62)	68 (7.08)	33 (3.43)	4 (0.42)
26	113 (50.45)	80 (35.71)	18 (8.036)	10 (4.46)	3 (1.33)
27	417 (59.66)	213 (30.47)	35 (5)	26 (3.72)	8 (1.15)
28	687 (57.59)	295 (24.73)	112 (9.39)	76 (6.37)	23 (1.93)
29	302 (57.52)	135 (25.71)	56 (10.67)	19 (3.62)	13 (2.48)
Z	12,915 (50.58)	4347 (17.03)	4975 (19.49)	2588 (10.14)	708 (2.77)
Total	133,031	38,813	34,582	16,229	3762
Percentage	58.75%	17.14%	15.27%	7.17%	1.66%

For the autosomal chromosomes of Mallard ducks, the percentage of dinucleotide repeats ranged between 13.97 (chr# 33) and 66.96% (chr# 6), the percentage of trinucleotide repeats ranged between 15.88 (chr# 8) and 65.52% (chr# 33), the percentage of tetranucleotide repeats ranged between 2.56 (chr# 31) and 17.80% (chr# 1), percentage of pentanucleotide repeats ranged between 3.45 (chr# 33) and 15.39% (chr# 31), and hexanucleotide repeats were not detected in chromosome 31 (0.00%) and reached the maximum percentage in chromosome 16 (9.02%).

For Muscovy duck (Table 3), the percentage of dinucleotide ranged from 69.08% (chr# 13) and 50.45% (chr# 26), while the trinucleotide calculated 35.71% (chr# 26) and 15.96 (chr#7), and the tetranucleotide scored ratio between 17.64% (chr# 1) and 3.21% (chr# 23). However, pentanucleotide repeats ranged between 8.39% (chr# 1) and 3.6% (chr# 22), and the hexanucleotide has means vary from 2.48% (chr# 29) and 0.42% (chr# 25).

Table 4 shows the size (Mb), relative abundance, and estimated repeat density of different chromosomes in Mallard and Muscovy ducks. For Mallards, chromosome 1 is the largest in size, followed by chromosomes 2 and 3, while the smallest are chromosomes 33, 32, and 17. Also, the reference sequence of mitochondrial DNA is too small (0.02 Mb). The highest relative microsatellite abundance was obtained in the sex chromosome (chromosome Z), which was 326.138, while the lowest relative abundance was scored for chromosome 20 and was 124.310. As also shown in Table 3, the estimated SSR density for Mallard chromosomes was low, and the lowest value was obtained for the Z chromosome. However, the highest estimated SSR density was obtained for mitochondrial DNA.

**Table 4:** Chromosome size, the total number, relative abundance, estimated repeat density of the detected microsatellite loci, and the number of SSR with primers in Mallard and Muscovy duck genomes

	Mallard					Muscovy				
Chr	Size (MB)	Total SSR	Relative abundance	Estimated repeat density	Total and % SSR with primers	Size (Mb)	Total SSR	Relative abundance	Estimated repeat density	Total and % SSR with primers
1	207.24	56,952	274.8118	0.001239	55,008 (96.6)	194.81	51,826	266.0336	0.001321	50,262 (96.98)
2	164.86	43,362	263.0232	0.001302	42,044 (96.96)	151.52	38,609	254.8112	0.001393	37,574 (97.32)
3	120.09	29,553	246.0904	0.001402	28,181 (95.4)	119.84	27,936	233.1108	0.001539	27,185 (97.31)
4	76.27	16,474	215.9958	0.001699	16,010 (97.2)	77.35	16,430	212.4111	0.001763	16,059 (97.74)
5	66.86	12,602	188.4834	0.001969	12,234 (97.1)	75.31	13,631	180.9985	0.002103	13,355 (97.98)
6	37.94	6398	168.6347	0.002309	6249 (97.7)	20.76	3598	173.3141	0.002221	3529 (98.08)
7	39.9	7070	177.193	0.002176	6871 (97.2)	29.88	5025	168.1727	0.00234	4926 (98.03)
8	32.62	5719	175.3219	0.002169	5588 (97.7)	49.68	8366	168.3977	0.002328	8172 (97.68)
9	26.79	4233	158.0067	0.00241	4148 (97.991)	28.78	4652	161.64	0.002388	4567 (98.17)
10	22.48	3744	166.548	0.002265	3651 (97.5)	19.93	3195	160.3111	0.002455	3124 (97.78)
11	22.6	3359	148.6283	0.002561	3275 (97.5)	17.05	2372	139.1202	0.002789	2316 (97.64)
12	21.61	3580	165.664	0.00228	3476 (97.1)	13.53	2289	169.1796	0.00231	2243 (97.99)
13	23.15	3535	152.6998	0.002589	3456 (97.8)	22.88	3441	150.3934	0.002669	3363 (97.73)
14	20.98	3379	161.0582	0.002139	3172 (93.9)	17.95	2543	141.6713	0.002675	2504 (98.47)
15	17.87	2514	140.6827	0.002755	2445 (97.3)	18.28	2490	136.2144	0.002881	2417 (97.07)
16	16.22	2638	162.6387	0.002105	2567 (97.3)	13.85	1969	142.1661	0.002792	1912 (97.11)
17	1.4	273	195	0.001607	267 (97.8)	0.47	118	251.0638	0.001378	107 (90.68)
18	12.07	1599	132.4772	0.002892	1544 (96.6)	12.34	1593	129.0924	0.003017	1511 (94.85)
19	13.13	1813	138.0807	0.002668	1735 (95.7)	11.89	1481	124.5585	0.00311	1430 (96.56)
20	11.97	1488	124.3108	0.003088	1436 (96.5)	12.09	1106	91.48056	0.004096	1019 (92.13)
21	16.96	2449	144.3986	0.00258	2378 (97.1)	16.47	2372	144.0194	0.002628	2329 (98.19)
22	8.4	1131	134.6429	0.002914	1099 (97.2)	8.55	1082	126.5497	0.003149	1058 (97.78)
23	5.33	721	135.272	0.002762	688 (95.4)	2.37	280	118.1435	0.003233	270 (96.43)
24	7.64	1144	149.7382	0.002428	1092 (95.5)	6.97	878	125.9684	0.003006	858 (97.72)
25	7.66	988	128.9817	0.003037	969 (98.1)	7.69	961	124.9675	0.003204	941 (97.92)
26	3.04	549	180.5921	0.001698	494 (89.98)	1.5	224	149.3333	0.002475	215 (95.98)
27	6.8	895	131.6176	0.002843	861 (96.2)	6.08	699	114.9671	0.003395	673 (96.28)
28	7.03	1189	169.1323	0.002036	1129 (94.95)	8.06	1193	148.0149	0.002499	1152 (96.56)
29	6.03	794	131.675	0.002767	752 (94.7)	4.02	525	130.597	0.002859	489 (93.14)
31	0.21	39	185.7143	0.00178	32 (82.1)					
33	0.11	29	263.6364	0.001146	27 (93.1)					
W	16.69	2264	135.6501	0.002618	2257 (99.7)					
Z	84.55	27,575	326.1384	0.000989	26,089 (94.6)	82.38	25,533	309.9417	0.001085	24,398 (95.56)
MT	0.02	1	50	0.01	1 (100)					

For Muscovy ducks, chromosome 1 is the largest one in size, followed by chromosomes 2 and 3 as found in Mallards, while chromosomes 17, 26, and 23 are the smallest respectively. The relative abundance of microsatellites varied from 309.941 in the Z chromosome to 91.481 in chromosome 20. The estimated SSR density for Muscovy chromosomes with the Z chromosome is having the lowest, while chromosome 20 had the highest estimated SSR density.

A large number of markers were designed for the detected markers for Mallards and Muscovies at the different chromosomes (Table 4). It can be observed for Mallards that the total SSR equals 250,053. The percentages of SSR markers with primers ranged between 100% for mitochondrial DNA to 82.1% at chromosome 31, while the percentage of SSR markers without primes was very low and reached its maximum at chromosome 31 with a percentage of 17.95% and its minimum in W chromosome with a percentage of 0.3%. The total SSR for Muscovies at different chromosomes equals 226,417. The percentages of SSR markers with primers ranged between 98.47% at chromosome 14 to 90.68% at chromosome 17. Similar to the results obtained for Mallards, the percentage of SSR markers without primes was also very low and reached maximum at chromosome 17 with a percentage of 7.87% and minimum at chromosome 14 with a percentage of 1.53%.

### Whole-genome SSR mining

When the whole genomes were analyzed, the results did not greatly differ from the chromosome-wise approach (Table 5), where dinucleotide motifs were predominant in both Mallard and Muscovy genomes and accounted for more than 50% of the detected motifs. However, the percentage was higher in Muscovy (58.39%) than that in Mallards (54.75%). Trinucleotide motifs ranked second among the two duck species and accounted for 17.61 and 16.87% of Muscovy and Mallard ducks. The tetranucleotide percentage was higher in Mallards (15.58%) than in Muscovy genomes. A similar trend was observed for pentanucleotides and hexanucleotides.

**Table 5:** Frequency and distribution of the microsatellite loci, the total number of SSR, relative abundance, estimated repeat density, and the number of SSR with primers in Mallard and Muscovy duck genomes using whole-genome mining approach

Species	Motif (*k*-mer)				
	**Di**	**Tri**	**Tetra**	**Penta**	**Hexa**
**Mallard**	142,386 (54.75)	43,862 (16.87)	40,513 (15.58)	25,208 (9.69)	8090 (3.11)
**Muscovy**	139,227 (58.39)	41,992 (17.61)	36,028 (15.11)	17,187 (7.21)	4028 (1.69)
	**Size (Mb)**	**Total SSR**	**Relative abundance**	**Estimated repeat density**	**Total SSR with primers**
**Mallard**	1188.52	260,059	218.8091	0.001578	250,657 (96.39)
**Muscovy**	1118.56	238,462	213.1866	0.001698	230,962 (96.86)

The size of the analyzed sequence of the Mallard duck genome was 1188.52 Mb, with a relative abundance equal to 218.81 and a low estimated repeat density of approximately 0.001578, while the total SSR counted 260,059, 96.39% of them found with primers. However, the analyzed sequence of the Muscovy genome was 1118.56 Mb, with a relative abundance equal to 218.81, and a low estimated repeat density of approximately 0.001698, while the total SSR counted 238,462, 96.86% of them found with primers.

As mentioned before, the number of detected microsatellites was higher in Mallards than in Muscovy ducks. Also, relative abundance was higher in Mallards than Muscovy (218.81 vs. 213.19). However, the estimated repeat density was higher in Muscovy than in Mallard ducks. Primers were designed for most of the generated microsatellites, with a percentage of 96.86 and 96.39% of the total microsatellites in Muscovy and Mallards, respectively.

### SSRs validation

The validation step was performed using 13 primer pairs for each of the Mallard and Muscovy ducks. All 26 microsatellites showed successful amplification of the genomes (Table 6). The genome amplification resulted in 122 alleles in Mallard and 119 alleles in Muscovy ducks. The number of the detected alleles per primer ranged between 0 and 9 alleles. The average number of alleles was 5.08 and 4.96 in Mallard and Muscovy ducks, respectively. Out of the 26 markers, one marker was monomorphic in Mallards, and two were monographic in Muscovy ducks. The number of private alleles was also higher in Mallards and reached 41 alleles, compared with 36 private alleles produced for the Muscovy duck genome. Overall primers, the minor allele frequency (MAF) averaged 0.31 and 0.36 in Mallard and Muscovy ducks, respectively. The average expected heterozygosity was slightly higher in Muscovy than in Mallards (0.45 vs. 0.43). The effective number of alleles was also higher in Muscovy (1.88 alleles) than in Mallards (1.81 alleles). The average polymorphic information content was also higher in Muscovy (0.55) than in Mallards (0.47). Table 7 shows the compression between primer sets generated for each species. No significant differences (*p* > 0.05) in the estimated parameters of the generated alleles were observed between primers designed for Muscovy and Mallard ducks.

**Table 6:** Observed allele range, number of alleles, number of private alleles, number of polymorphic alleles, and number of monomorphic alleles

Marker ID	Species	Observed allele range	Number of alleles		The number of private alleles		The number of polymorphic alleles		The number of Monomorphic alleles	
			Mallard	Muscovy	Mallard	Muscovy	Mallard	Muscovy	Mallard	Muscovy
MK0022	Muscovy	300–350	4	6	2	2	2	3	2	3
MK37651	Muscovy	233–452	7	8	3	3	5	8	2	0
MK37669	Muscovy	260–430	6	6	1	2	6	5	0	1
MK37680	Muscovy	355–432	6	7	1	3	0	3	6	4
MK19696	Muscovy	310–364	3	4	0	1	3	3	0	1
MK19508	Muscovy	323–423	4	4	1	1	3	1	1	3
MK19002	Muscovy	234–293	2	7	0	2	2	5	0	2
MK38645	Muscovy	158–233	5	6	3	2	3	5	2	1
MK33624	Muscovy	200–380	5	4	2	2	5	3	0	1
MK31272	Muscovy	153–333	3	4	3	2	3	4	0	0
MK26001	Muscovy	141–283	6	4	1	0	4	3	2	1
MK37601	Muscovy	165–378	7	6	2	1	4	2	3	4
MK46014	Mallard	280–470	7	7	3	2	6	7	1	0
MK4647	Mallard	214–333	5	4	2	2	4	2	1	2
MK5039	Mallard	320–440	3	5	1	1	2	3	1	2
MK4624	Mallard	232–454	5	4	1	0	3	4	2	0
MK24903	Mallard	143–381	8	6	4	2	8	5	0	1
MK25130	Mallard	221,434	9	6	3	2	3	5	6	1
MK25365	Mallard	190–355	2	4	2	3	2	3	0	1
MK3631	Mallard	156–273	5	5	1	1	4	2	1	3
MK8745	Mallard	210–344	3	3	0	0	1	0	2	3
MK11161	Mallard	140–352	6	2	2	0	3	2	3	0
MK17028	Mallard	165–266	5	3	1	1	2	0	3	3
MK15735	Mallard	278–465	6	4	2	1	5	1	1	3
Total		141–465	122	119	41	36	83	79	39	40
Mean			5.08	4.96	1.71	1.5	3.46	3.29	1.63	1.67
Range			2–9	2–8	0–4	0–3	0–8	0–8	0–6	0–4

**Table 7:** Number of alleles, minor allele frequency, expected heterozygosity, number of effective alleles, and polymorphic information content of Mallard and Muscovy ducks and cross-translatability of the primers generated for the two species

Species	Primers	Number of alleles		Minor allele frequency		Expected heterozygosity		Number of effective alleles		Polymorphic information content	
		Mean	SD	Mean	SD	Mean	SD	Mean	SD	Mean	SD
Mallard	Muscovy primers	4.83	1.60	0.36	0.10	0.45	0.10	1.87	0.34	0.50	0.09
	Mallard primers	5.33	2.06	0.31	0.10	0.41	0.11	1.74	0.31	0.44	0.10
	*p* <	0.52		0.30		0.31		0.33		0.20	
Muscovy	Muscovy primers	5.50	1.50	0.54	0.10	0.46	0.10	1.91	0.31	0.52	0.10
	Mallard primers	4.12	1.40	0.55	0.83	0.45	0.08	1.85	0.29	0.59	0.77
	*p* <	0.08		0.71		0.71		0.65		0.06	

## Discussion

Microsatellites are simple, tenderly repeated sequence motifs flanked by unique sequences. Over the past two decades, microsatellites considered the marker of choice for animal breeders due to their polymorphic and codominant nature. They have been used in the studies of diversity, genome mapping, and evolutional and ecological genetics [19, 20]. Also, they have been incorporated into marker-assisted selection programs [21]. Different search tools were developed for in silico mining of microsatellite repeats from assembled genome sequences. However, most animal genomes were not subjected to that analysis; this may be due to the large size of the sequenced genomes of the different animals, concurrently with the fact that most of the analysis tools were designed to analyze short sequences. Accordingly, we used the GMATA application due to the possibility of analyzing whole-genome sequences and due to the possibility of analyzing whole genomes. We examined and screened the distribution of microsatellite loci in Mallard and Muscovy ducks, using chromosome-wise and whole-genome approaches. This was performed using the reference sequences available in NCBI, where the analysis resulted in several noteworthy findings.

Muscovy and Mallard ducks belong to the same family Anatidae, but Muscovy is more land oriented than Mallard. The two duck species exhibit different phenotypic and genotypic variations. Moreover, Muscovy and Mallard ducks are genetically different [22]. Muscovy has a higher proportion of large chromosomes compared to the Mallard duck, which suggests that it may have experienced more chromosomal rearrangements during its evolution [23].

During the past two decades, there have been different attempts to develop microsatellite markers for ducks, starting in the year 2000 with the development of seven microsatellites [24]. Also, Huang et al. [25] developed 35 markers, where 28 of them were polymorphic. Recently, Zhang et al. [26] developed 24 markers for Jinding ducks. All the previous studies were directed to develop a limited number of microsatellites due to the high detected costs. To our knowledge, this is the first report about the whole-genome comparison of microsatellite distribution in Mallard and Muscovy ducks.

Among the detected microsatellite loci in both Muscovy and Mallard genomes, the dinucleotide repeats were the most abundant (as shown in Tables 2 and 3); this is in agreement with that reported for most vertebrates [27–29]. The reductions in microsatellite abundance with increasing the length of repeats were previously documented [28, 30, 31]. Fan et al. [31] reported that the distribution of microsatellites in the duck genome is not random and may be influenced by the selection pressure. The high percentage of short motifs suggests that there is some evolutionary advantage to having a high frequency of dinucleotide and trinucleotide repeats, such as increased genetic stability or more efficient gene regulation. Furthermore, previous studies have demonstrated that larger chromosomes generally have higher relative abundances in other organisms as well [32, 33], suggesting that this relationship may be generalizable across species [34]. For the same reason, the size and total detected microsatellites in Muscovy and Mallard chromosomes have been found to be related to their relative abundance. Specifically, the larger the size of a chromosome, the greater its relative abundance [35]. This is attributed to the fact that larger chromosomes tend to contain more SSRs than smaller chromosomes.

Size and total SSR are two important parameters that can be used to compare the relative abundance of Muscovy and Mallard chromosomes and genomes (Table 4). Muscovy has larger chromosomes than Mallard, but its genome size is much smaller. On the other hand, Mallard has smaller chromosomes but a larger genome size [36, 37]. The total SSR content of Muscovy is lower than that of Mallard, indicating that the relative abundance of SSRs in Muscovy is lower than in Mallard. This may be the main reason why the relative abundance of SSRs in Muscovy is less than that in Mallard, which could be due to differences in genome organization or gene content between these two species [38]. Furthermore, the higher SSR content of Mallard compared to Muscovy could also be attributed to its larger genome size, which allows for more genetic variation. Thus, size and total SSR content can be used to compare the relative abundance of chromosomes and genomes between species [39, 40].

In the current study, 26 primer pairs for both Muscovies and Mallards were randomly selected for validation of the detected loci and the designed primers (Table 1). All primers produced clear stable bands. The number of the detected bands was higher in Muscovy than in Mallard ducks (Table 7). This is in agreement with the results of [41], who reported an average number of detected alleles of 2.44 and 2.20 in Muscovy and Mallard ducks, respectively. Huang et al. [25] developed 35 SSRs for Peking ducks and detected 117 different alleles, which is close to our results (119 and 122 alleles in Muscovies and Mallards). A higher number of alleles (192 alleles) was detected by Wu et al. [42], inbred by 18 microsatellite markers. Ahmadi et al. [41] used 13 markers to compare Muscovy and Pekin (Mallard) ducks and reported a higher average of allelic number in Muscovy (2.4 alleles) compared to Pekin (2.2 alleles).

The expected heterozygosity results were moderate and ranged between 0.22 and 0.58 (Table 7). Similar results were obtained previously [41, 42]. A wider range from 0.02 to 0.98 was estimated previously [25]. Contrary to our results, it was also reported higher expected heterozygosity in Mallards (0.43) compared to Muscovy (0.41); however, the values are similar to our results that averaged 0.44. The average PIC values of the markers used for the current study were close to those observed for the 28 markers that were developed by Huang [25]. A similar value of 0.46 was reported previously as an average of PIC values for 22 microsatellites [43].

Cross-species microsatellite markers provide a powerful tool for studying genetic diversity and hybridization events between different species and can help inform conservation efforts for endangered species. This approach involves utilizing species-specific microsatellites by means of cross-species amplification, which relies on the high relatedness of species and does not entail extra expenses [44–46]. In such cases, the most favorable results are achieved by amplifying different fragments of species that share the same genus or belong to closely related genera. Hence, the efficacy of cross-amplifying any DNA sequence is inversely proportional to the evolutionary divergence between two species [46]. For these reasons, we also examined the cross-species transferability SSR markers between Muscovy and Mallard ducks. The results denoted the usefulness transferability of makers between Mallards and Muscovies, where the differences between the two duck species in all parameters were insignificant indicating that 100% of the primers designed for the Mallards could be useful in the analysis of the Muscovy genome and vice versa. Huang et al. [25] screened the cross-species amplification of duck microsatellites by screening the polymorphism in chickens and geese, where two duck markers produced monomorphic alleles in chickens, and 14 makers amplified the geese genome, and the differences were attributed to the different genetic distances among the testes species.

The current study identified and characterized SSRs in the genome of Mallard and Muscovy ducks, which are very important and have diverse applications, such as their application in population genetics studies [26], identifying markers that are associated with different genes and traits [47], assessing genetic diversity [48], development of linkage maps [5], and studying secondary structure formation [49]. In addition, comparing the distribution and density of microsatellites is important for evolutionary studies [50].

## Conclusion

The present study investigated the distribution and frequency of microsatellites in the genomes of Mallard and Muscovy ducks. Two approaches were used to achieve this objective, and the findings revealed minimal differences between the two methods. However, the genome-wide in silico microsatellite mining approach was observed to be relatively faster than the chromosome-wise approach. The results also demonstrated the potential of cross-species amplification of different primer sets between Mallard and Muscovy ducks. The significant number of developed microsatellite loci identified in this study is expected to serve as a valuable resource for mapping QTLs, assessing genetic diversity and population structure, and facilitating marker-assisted breeding applications. Further efforts should be directed towards identifying microsatellites located in the EST regions and towards predicting the coding and function of the identified microsatellites.

## Data Availability

Supporting data files which include markers developed for Mallard and Muscovy ducks are freely available to access at https://github.com/mosthamed/DUCK_SSR_primers.
